# Association between gamma-glutamyl transferase and risk of major adverse cardiovascular events in patients with hypertrophic cardiomyopathy: a retrospective cohort study

**DOI:** 10.3389/fcvm.2026.1900684

**Published:** 2026-08-28

**Authors:** Meng Yao, Xiaoyin Xing, Zhe Chen, Cunying Cui, Xinqiao Tian

**Affiliations:** Department of Ultrasound, Fuwai Central China Cardiovascular Hospital, Central China Fuwai Hospital of Zhengzhou University, Zhengzhou, China

**Keywords:** cohort study, gamma-glutamyl transferase, hypertrophic cardiomyopathy, major adverse cardiovascular events, risk

## Abstract

**Background:**

Hypertrophic cardiomyopathy (HCM) is the most common hereditary heart disease, and the subsequent major adverse cardiovascular events (MACE) pose a serious threat to the health of patients. This investigation was intended to explore the association between gamma-glutamyl transferase (GGT) levels and MACE risk in patients with HCM.

**Methods:**

Data from patients (≥ 18 years old) diagnosed with HCM at a hospital were enrolled in this retrospective cohort study. The association between GGT levels and the risk of MACE was assessed using Cox regression models. Subgroup analyses were conducted according to HCM subtypes (non-obstructive, resting obstructive, latent obstructive) and New York Heart Association (NYHA) grades (I/II, III/IV).

**Results:**

The analysis cohort consisted of 437 patients with HCM, of whom 163 (37.30%) patients experienced MACE, while 274 (62.70%) remained MACE-free. GGT levels of 20–57 U/L [HR = 1.72 (95%CI: 1.13–2.62)] and ≥57 U/L [HR = 2.29 (95%CI: 1.30–4.03)] were both associated with a higher risk of MACE than those with GGT <20 U/L. Subgroup analysis found that GGT levels of 20–57 U/L [HR = 2.25 (95%CI: 1.15–4.37)] and ≥57 U/L [HR = 3.66 (95%CI: 1.44–9.28)] were both linked to an increased risk of MACE in those with non-obstructive HCM, whereas no significant association was detected in those with resting obstructive HCM and latent obstructive HCM. Moreover, GGT levels of 20–57 U/L [HR = 1.78 (95%CI: 1.07–2.97)] and ≥57 U/L [HR = 2.97 (95%CI: 1.51–5.83)] were both linked to an elevated risk of MACE in patients with NYHA grade I/II.

**Conclusion:**

Elevated GGT levels were associated with a higher risk of MACE among patients with HCM. GGT may serve as a clinical red flag for MACE risk.

## Introduction

Hypertrophic cardiomyopathy (HCM) is a prevalent hereditary heart disease characterized by left ventricular hypertrophy and absence of normal load conditions (1). Left ventricular hypertrophy and abnormal ventricular configuration often result in dynamic obstruction of the left ventricular outflow tract in the majority of patients (1). HCM exhibits significant heterogeneity, with its global prevalence estimated to be 0.5% (2). HCM may increase the risk of major adverse cardiovascular events (MACE), including sudden cardiac death (SCD), heart failure, and atrial fibrillation (2, 3). Approximately 5.3% to 15% of HCM patients develop MACE annually (4, 5), and about 90% of those diagnosed with HCM at a young age (≤40 years old) will have MACE over the course of their lifetime (6). The clinical outcomes of patients with HCM are influenced not only by anatomical structures and hemodynamic changes but also by multiple mechanisms, including oxidative stress, inflammatory responses, myocardial fibrosis, and metabolic remodeling (7–9).

Gamma-glutamyl transferase (GGT) is a key enzyme in the glutathione metabolism process, which participates in the decomposition and recycling of extracellular glutathione and plays a crucial role in maintaining redox homeostasis (10, 11). Elevated GGT levels are associated with oxidative stress, low-grade chronic inflammation, metabolic abnormalities, and atherosclerotic processes (11, 12), all of which play a significant role in the development of cardiovascular disease and its impact on mortality. N-terminal pro-B-type natriuretic peptide (NT-proBNP) and high-sensitivity troponin reflect myocardial wall stress and myocardial injury, respectively, and their roles in assessing the risk of HCM have received considerable attention (13). However, the progression of HCM involves not only hemodynamic stress and myocardial damage but may also be accompanied by oxidative stress, inflammatory responses, and metabolic abnormalities (2). Compared with NT-proBNP and high-sensitivity troponin, there have been few studies investigating the impact of GGT levels, which are associated with oxidative stress, inflammatory responses, and metabolic abnormalities, on patients with HCM. Epidemiological evidence suggests that higher levels of GGT are linked to an increased risk of cardiovascular mortality, all-cause mortality, and various adverse cardiovascular outcomes (14–16). Elevated GGT serves as a valuable indicator for predicting subsequent cardiovascular events among patients with acute coronary syndromes (17). GGT levels are significantly correlated with subclinical coronary atherosclerosis and adverse cardiac events (18), and GGT variability acts as a strong predictor of heart failure hospitalization risk (19). Furthermore, GGT levels have also been reported to correlate with metabolic syndrome, obesity, and insulin resistance (20, 21). However, the relationship between GGT levels and the risk of MACE among patients with HCM remains unclear. Currently, risk assessment for HCM integrates clinical history, ambulatory electrocardiographic monitoring, echocardiography, and cardiac magnetic resonance imaging. Guideline-based approaches primarily focus on the risk of SCD and decisions regarding implantable cardioverter-defibrillators, while atrial fibrillation, heart failure progression, and functional deterioration are typically assessed separately (22, 23). Clinical prediction models such as the HCM Risk-SCD model have been established to estimate SCD risk among patients with HCM (24). However, MACE (i.e., myocardial infarction, heart failure, atrial fibrillation, and SCD) in HCM is a heterogeneous composite endpoint, and predictors of arrhythmic and non-arrhythmic outcomes, as well as SCD, may not adequately identify patients at high risk for hospitalization due to heart failure or other adverse cardiovascular events. Accordingly, readily available indicators that provide supplementary information on short- and medium-term MACE risk warrant further investigation. Thus, the present study was designed to explore the association between GGT levels and MACE risk in patients with HCM, and assess the prognostic value of GGT for predicting outcomes.

## Methods

### Participants and study design

The data for this retrospective cohort analysis were extracted from patients who visited the Ultrasound Department of Fuwai Central China Cardiovascular Hospital between April 2018 and July 2025. This study included patients aged 18 years or above who were diagnosed with HCM. The exclusion criteria applied to the patients were as follows: (1) Patients who underwent other cardiovascular procedures, such as coronary artery intervention therapy or aortic valve surgery; (2) Patients with missing GGT data; (3) Patients without echocardiography examination; and (4) Patients with a follow-up duration of less than 30 days or without any follow-up data. The diagnosis of HCM was based on the criteria of the AHA/ACC guidelines (2020): HCM is diagnosed when echocardiography or cardiac magnetic resonance reveals a left ventricular end-diastolic wall thickness of ≥15 mm at any site (25). If other characteristics are present, including a family history, a positive result for pathogenic gene testing, and electrocardiogram abnormalities, HCM can also be diagnosed according to a left ventricular wall thickness of ≥13 mm. Based on the changes in left ventricular outflow tract gradient (LVOTG), HCM is classified into non-obstructive (peak LVOTG < 30 mmHg at rest and after exercise), resting obstructive (peak LVOTG ≥ 30 mmHg at rest), and latent obstructive (peak LVOTG < 30 mmHg at rest but ≥ 30 mmHg after exercise) (26). This study was approved by the Ethics Review Committee of Fuwai Central China Cardiovascular Hospital (No. 2025-138). As this was a retrospective study, informed consent from patients was not required.

### Outcome and exposure

The occurrence of MACE in patients with HCM during follow-up was the endpoint of this study. MACE includes SCD, hospitalization for heart failure, new-onset stroke, heart transplantation, sustained ventricular tachycardia or ventricular fibrillation, and all-cause mortality. The date of index admission was defined as the starting point of follow-up. Baseline serum GGT measurement and transthoracic echocardiography were completed within 1 day of admission, with the time interval between baseline testing and the start of follow-up not exceeding 1 day. Follow-up data were obtained from outpatient follow-up records, readmission medical records, and telephone interviews. Patients were followed up from the start of this hospitalization until the first occurrence of a predefined MACE or until the study cutoff date (January 2026). Additionally, to ensure adequate observational duration for clinical outcomes, reduce incomplete outcome information caused by extremely short follow-up periods, and minimize prognostic confounding attributable to unstable early in-hospital conditions and ongoing treatment adjustments during the index hospitalization, patients with a follow-up duration of fewer than 30 days were excluded from the final analysis.

GGT levels served as the exposure variable in this analysis. Fasting blood samples were collected from the patients, and the serum sample was used for GGT detection through an automatic Cobas-c6000 biochemical analyzer (Roche Diagnostics, Mannheim, Germany). Serum GGT levels were determined using an enzymatic colorimetric method (Roche Modular P; Roche Diagnostics, Mannheim, Germany). The hospital clinical laboratory implemented twice-daily quality control for GGT assays, conducted at instrument startup each morning and shutdown each evening. Target values were aggregated monthly by the laboratory to establish laboratory-specific quality control ranges for GGT testing. GGT measurements were routine clinical measurements retrieved from the medical records. The X-title was employed to determine the cutoff values for GGT levels. X-title evaluated possible cutoff combinations and selected the values that yielded the maximum log-rank *χ*² statistic, which corresponds to the scenario with the greatest difference in outcomes between groups. Two cutoff values, 20 and 57 U/L, were ultimately determined, and GGT levels were divided into three groups: low (<20 U/L), median (20–57 U/L), and high (≥57 U/L) (Supplementary Figure S1).

### Covariates

Relevant data from patients were collected, including age, gender (male, female), body mass index (BMI: <24, ≥24 kg/m^2^), smoking status (no, quit smoking, current smoking), drinking status (no, quit drinking, current drinking), time science diagnosis, family history of HCM (no, yes), family history of SCD (no, yes), unexplained syncope within the past 6 months (no, yes), history of cardiac surgery (no, yes), history of HCM-related surgery (no, yes), chest tightness (no, yes), palpitations (no, yes), chest pain (no, yes), hypertension (no, yes), diabetes (no, yes), dyslipidemia (no, yes), coronary heart disease (no, yes), atrial fibrillation (no, yes), New York Heart Association (NYHA) grade (I/II, III/IV), white blood cell count (WBC), lymphocyte, hemoglobin, platelet count, alanine aminotransferase (ALT), aspartate aminotransferase (AST), albumin, total bilirubin, alkaline phosphatase, anion gap, plasma colloid osmotic pressure (PCOP), glucose, estimate glomerular filtration rate (eGFR), triglyceride, total cholesterol, high-density lipoprotein cholesterol (HDL-C), low-density lipoprotein cholesterol (LDL-C), NT-proBNP, international normalized ratio (INR), fibrinogen, non-sustained ventricular tachycardia (no, yes), HCM subtype (non-obstructive, resting obstructive, latent obstructive), maximum left ventricular wall thickness, left atrial diameter, left ventricular end-systolic diameter, left ventricular end-diastolic diameter, mitral inflow E wave to A wave ratio, mitral E wave to tissue Doppler e ratio, left ventricular ejection fraction, left ventricular apical aneurysm (no, yes), treatment strategy (no treatment, intervention only, surgery only), implantable cardioverter-defibrillator (no, yes), beta-blockers (no, yes), calcium channel blockers (no, yes), angiotensin-converting enzyme inhibitors/angiotensin II receptor blockers (ACEI/ARB: no, yes), diuretics (no, yes), antiarrhythmic drugs (no, yes), mineralocorticoid receptor antagonists (no, yes), antiplatelet therapy (no, yes), statins (no, yes), and follow-up time.

### Statistical analysis

Missing data were observed in some covariates, and multiple imputation was employed to impute these missing values. Comparisons of these variables were conducted between pre-imputation and post-imputation datasets (Supplementary Table S1). Continuous variables were described as mean ± standard deviation (SD) or median with interquartile range [M (Q1, Q3)]. ANOVA or Kruskal–Wallis H test was performed to compare differences among groups. Categorical variables were summarized as numbers and proportions [n (%)], with group differences analyzed by chi-square test or Fisher's exact test.

Univariate Cox regression analysis was employed to screen for confounders associated with MACE risk, and variables showing *P* < 0.05 in this univariate model were incorporated into the multivariate model for adjustment (Supplementary Table S2). Cox regression models were employed to evaluate the association between GGT levels and the risk of MACE among patients with HCM, with the association presented as hazard ratio (HR) and 95% confidence interval (CI). Model 1 was a univariate Cox regression model. Model 2 was adjusted for age, history of cardiac surgery, alkaline phosphatase, eGFR, non-sustained ventricular tachycardia, and implantable cardioverter-defibrillator. Model 3 was further adjusted for all clinically significant variables (HCM subtype, left ventricular ejection fraction, NT-proBNP, maximum left ventricular wall thickness, left atrial diameter, family history of SCD, non-sustained ventricular tachycardia, unexplained syncope within the past 6 months, history of HCM-related surgery) based on model 2. The Kaplan–Meier was applied to evaluate the impact of different GGT levels on MACE-free survival. The relationship between GGT levels and MACE risk was further explored in HCM subtype (non-obstructive, resting obstructive, latent obstructive) and NYHA grade (I/II, III/IV) subgroups. The Cox regression model, which contained age, history of cardiac surgery, alkaline phosphatase, eGFR, non-sustained ventricular tachycardia, implantable cardioverter-defibrillator, and GGT, was applied to predict 1-year and 2-year MACE risk. The predictive performance of the model was assessed by the area under the curve (AUC), Net Reclassification Index (NRI), and Integrated Discrimination Improvement (IDI). Statistical analyses were performed using SAS 9.4 software (SAS Institute Inc., Cary, NC, USA), while graphical visualization of predictive models was generated with R 4.5.1 software (Institute for Statistics and Mathematics, Vienna, Austria). Statistical significance was set at a threshold of *P* < 0.05.

## Results

### Participant characteristics

**Figure 1:**
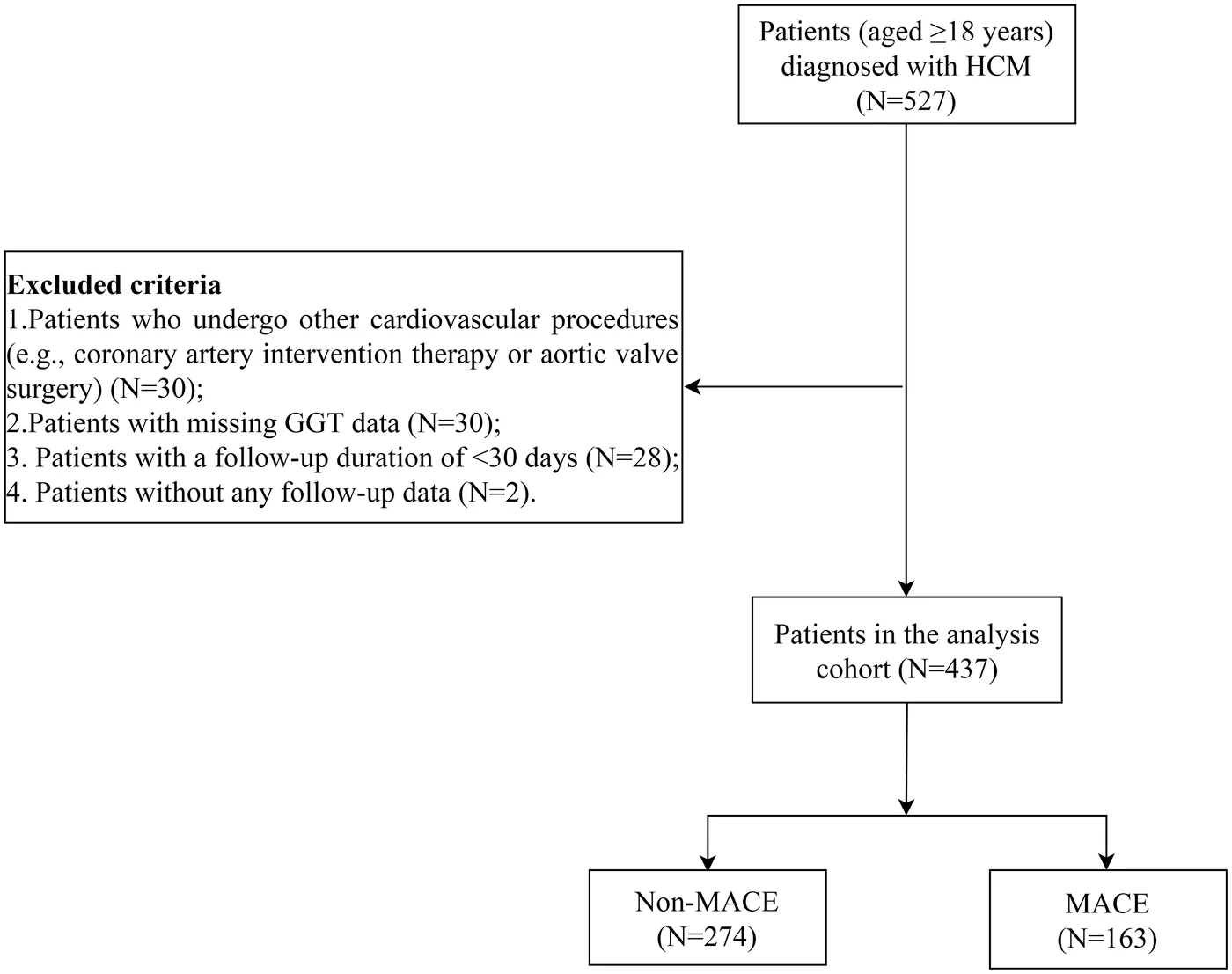
Flowchart for patient screening. HCM, hypertrophic cardiomyopathy; GGT, gamma-glutamyl transferase; MACE, major adverse cardiovascular events.

A total of 527 adult patients diagnosed with HCM were selected. Following screening, 90 patients were excluded, and the remaining 437 patients were enrolled in the analysis cohort (Figure 1). Among the patients with HCM, 163 (37.30%) experienced MACE, whereas 274 (62.70%) remained MACE-free. The components of MACE were distributed as follows: SCD (1 case, 0.23%), heart failure hospitalization (151 cases, 34.55%), new-onset stroke (7 cases, 1.60%), sustained ventricular tachycardia or ventricular fibrillation (2 cases, 0.46%), and all-cause mortality (13 cases, 2.97%). The median follow-up time was 741.00 (359.00, 1039.00) days. Table 1 summarizes the characteristics of patients in different GGT level groups. In terms of GGT level distribution, 160 (36.61%) patients had GGT <20 U/L, 212 (48.51%) had GGT ranging from 20 to 57 U/L, and 65 (14.88%) had GGT ≥57 U/L. The mean age was 54.86 ± 12.88 years, and 271 (62.01%) patients were male. According to HCM classification, 207 (47.37%) patients had non-obstructive HCM, 194 (44.39%) patients had resting obstructive HCM, and 36 (8.24%) patients had latent obstructive HCM. Patients in different GGT level groups showed significant differences in age, gender, smoking status, drinking status, time since diagnosis, family history of HCM, atrial fibrillation, WBC, hemoglobin, platelet count, ALT, AST, total bilirubin, alkaline phosphatase, triglyceride, total cholesterol, LDL-C, left atrial diameter, and mitral E wave to tissue Doppler e ratio.

**Table 1:** Characteristics of patients with HCM.

Variables	Total (*n* = 437)	Gamma-Glutamyl Transferase (U/L)			*P*
		<20 (*n* = 160)	20-57 (*n* = 212)	≥57 (*n* = 65)	
Age (year), Mean ± SD	54.86 ± 12.88	58.69 ± 11.77	53.18 ± 13.08	50.89 ± 12.67	<0.001
Gender, *n* (%)					<0.001
Male	271 (62.01)	75 (46.88)	146 (68.87)	50 (76.92)	
Female	166 (37.99)	85 (53.13)	66 (31.13)	15 (23.08)	
BMI (kg/m2), Mean ± SD	26.72 ± 3.79	26.42 ± 3.65	26.93 ± 3.74	26.77 ± 4.30	0.434
BMI (kg/m2), *n* (%)					0.211
<24	100 (22.88)	37 (23.13)	43 (20.28)	20 (30.77)	
≥24	337 (77.12)	123 (76.88)	169 (79.72)	45 (69.23)	
Smoking status, *n* (%)					<0.001
No	229 (52.40)	107 (66.88)	96 (45.28)	26 (40.00)	
Quit smoking	96 (21.97)	27 (16.88)	52 (24.53)	17 (26.15)	
Current smoking	112 (25.63)	26 (16.25)	64 (30.19)	22 (33.85)	
Drinking status, *n* (%)					<0.001
No	230 (52.63)	110 (68.75)	97 (45.75)	23 (35.38)	
Quit drinking	109 (24.94)	30 (18.75)	61 (28.77)	18 (27.69)	
Current drinking	98 (22.43)	20 (12.50)	54 (25.47)	24 (36.92)	
Time since diagnosis (days), M (Q_1_, Q_3_)	31.00 (0.00, 743.00)	41.50 (0.00, 908.00)	30.00 (0.00, 758.00)	2.00 (0.00, 196.00)	0.089
Family history of HCM, *n* (%)					0.042
No	418 (95.65)	156 (97.50)	204 (96.23)	58 (89.23)	
Yes	19 (4.35)	4 (2.50)	8 (3.77)	7 (10.77)	
Family history of SCD, *n* (%)					0.638
No	431 (98.63)	157 (98.13)	210 (99.06)	64 (98.46)	
Yes	6 (1.37)	3 (1.88)	2 (0.94)	1 (1.54)	
Unexplained syncope within the past 6 months, *n* (%)					0.375
No	400 (91.53)	143 (89.38)	198 (93.40)	59 (90.77)	
Yes	37 (8.47)	17 (10.63)	14 (6.60)	6 (9.23)	
History of cardiac surgery, *n* (%)					0.589
No	280 (64.07)	103 (64.38)	132 (62.26)	45 (69.23)	
Yes	157 (35.93)	57 (35.63)	80 (37.74)	20 (30.77)	
History of HCM-related surgery, *n* (%)					0.114
No	353 (80.78)	122 (76.25)	174 (82.08)	57 (87.69)	
Yes	84 (19.22)	38 (23.75)	38 (17.92)	8 (12.31)	
Chest tightness, *n* (%)					0.910
No	191 (43.71)	69 (43.13)	92 (43.40)	30 (46.15)	
Yes	246 (56.29)	91 (56.88)	120 (56.60)	35 (53.85)	
Palpitations, *n* (%)					0.727
No	333 (76.20)	123 (76.88)	163 (76.89)	47 (72.31)	
Yes	104 (23.80)	37 (23.13)	49 (23.11)	18 (27.69)	
Chest pain, *n* (%)					0.539
No	386 (88.33)	141 (88.13)	185 (87.26)	60 (92.31)	
Yes	51 (11.67)	19 (11.88)	27 (12.74)	5 (7.69)	
Hypertension, *n* (%)					0.533
No	228 (52.17)	87 (54.38)	111 (52.36)	30 (46.15)	
Yes	209 (47.83)	73 (45.63)	101 (47.64)	35 (53.85)	
Diabetes, *n* (%)					0.285
No	402 (91.99)	143 (89.38)	199 (93.87)	60 (92.31)	
Yes	35 (8.01)	17 (10.63)	13 (6.13)	5 (7.69)	
Dyslipidemia, *n* (%)					0.716
No	410 (93.82)	151 (94.38)	197 (92.92)	62 (95.38)	
Yes	27 (6.18)	9 (5.63)	15 (7.08)	3 (4.62)	
Coronary heart disease, *n* (%)					0.562
No	277 (63.39)	99 (61.88)	133 (62.74)	45 (69.23)	
Yes	160 (36.61)	61 (38.13)	79 (37.26)	20 (30.77)	
Atrial fibrillation, *n* (%)					0.010
No	341 (78.03)	135 (84.38)	163 (76.89)	43 (66.15)	
Yes	96 (21.97)	25 (15.63)	49 (23.11)	22 (33.85)	
NYHA grade, *n* (%)					0.981
I/II	340 (77.80)	125 (78.13)	165 (77.83)	50 (76.92)	
III/IV	97 (22.20)	35 (21.88)	47 (22.17)	15 (23.08)	
WBC count (10^9^/L), Mean ± SD	6.21 ± 1.77	5.66 ± 1.61	6.43 ± 1.76	6.87 ± 1.84	<0.001
Lymphocyte count (10^9^/L), M (Q_1_, Q_3_)	1.70 (1.30, 2.20)	1.70 (1.30, 2.20)	1.70 (1.40, 2.20)	1.80 (1.40, 2.20)	0.153
Hemoglobin (g/L), M (Q_1_, Q_3_)	141.00 (127.00, 152.00)	133.00 (122.00, 145.00)	144.50 (131.50, 154.50)	149.00 (134.00, 156.00)	<0.001
Platelet count (10^9^/L), Mean ± SD	201.24 ± 59.04	191.61 ± 55.01	204.49 ± 58.80	214.40 ± 66.24	0.017
ALT (U/L), M (Q_1_, Q_3_)	19.00 (14.00, 29.00)	14.00 (11.00, 20.00)	21.00 (16.00, 30.50)	30.00 (20.00, 49.00)	<0.001
AST (U/L), M (Q_1_, Q_3_)	19.00 (16.00, 24.00)	18.00 (14.00, 21.00)	20.00 (16.00, 25.00)	24.00 (18.00, 34.00)	<0.001
Albumin (g/L), Mean ± SD	41.77 ± 4.69	41.19 ± 4.02	42.25 ± 5.12	41.68 ± 4.67	0.097
Total Bilirubin (μmol/L), M (Q_1_, Q_3_)	10.00 (8.00, 14.90)	10.00 (7.00, 13.00)	11.00 (8.00, 15.00)	11.00 (8.50, 15.00)	0.048
Alkaline Phosphatase (U/L), Mean ± SD	71.86 ± 19.96	68.82 ± 17.63	72.59 ± 20.26	76.95 ± 23.16	0.016
Anion gap (mmol/L), Mean ± SD	300.72 ± 12.17	301.17 ± 6.08	301.28 ± 5.46	297.76 ± 28.44	0.105
PCOP (mOsm/L), Mean ± SD	13.77 ± 2.95	13.41 ± 2.65	13.94 ± 3.13	14.12 ± 2.98	0.129
Glucose (mmol/L), M (Q_1_, Q_3_)	4.80 (4.30, 5.50)	4.70 (4.20, 5.40)	4.80 (4.30, 5.50)	4.90 (4.40, 5.90)	0.326
eGFR (mL/min), M (Q_1_, Q_3_)	83.00 (71.00, 95.40)	82.00 (68.00, 92.50)	84.00 (74.00, 96.00)	85.00 (71.00, 97.00)	0.245
Triglyceride (mmol/L), M (Q_1_, Q_3_)	1.30 (0.95, 1.90)	1.00 (0.80, 1.55)	1.40 (1.00, 1.90)	1.50 (1.10, 2.30)	<0.001
Total Cholesterol (mmol/L), Mean ± SD	3.93 ± 0.97	3.75 ± 0.95	4.02 ± 1.00	4.10 ± 0.88	0.012
HDL-C (mmol/L), M (Q_1_, Q_3_)	1.00 (0.80, 1.20)	1.00 (0.83, 1.20)	1.00 (0.89, 1.20)	0.97 (0.80, 1.10)	0.140
LDL-C (mmol/L), M (Q_1_, Q_3_)	2.30 (1.70, 2.90)	2.10 (1.60, 2.75)	2.40 (1.70, 3.00)	2.40 (2.00, 3.00)	0.016
NT-proBNP (pg/mL), M (Q_1_, Q_3_)	795.00 (299.00, 1,823.00)	786.00 (342.00, 1,785.00)	835.00 (311.00, 1,691.00)	678.00 (186.00, 2,574.00)	0.787
INR, M (Q_1_, Q_3_)	1.00 (0.90, 1.00)	1.00 (0.90, 1.00)	1.00 (0.90, 1.00)	0.90 (0.90, 1.00)	0.099
Fibrinogen (g/L), M (Q_1_, Q_3_)	2.60 (2.20, 3.10)	2.50 (2.20, 3.00)	2.60 (2.20, 3.20)	2.80 (2.35, 3.10)	0.063
Non-sustained ventricular tachycardia, *n* (%)					0.798
No	424 (97.03)	154 (96.25)	206 (97.17)	64 (98.46)	
Yes	13 (2.97)	6 (3.75)	6 (2.83)	1 (1.54)	
HCM subtype, *n* (%)					0.152
Non-obstructive	207 (47.37)	68 (42.50)	100 (47.17)	39 (60.00)	
Resting obstructive	194 (44.39)	79 (49.38)	95 (44.81)	20 (30.77)	
Latent obstructive	36 (8.24)	13 (8.13)	17 (8.02)	6 (9.23)	
Maximum left ventricular wall thickness (mm), Mean ± SD	21.75 ± 4.21	21.49 ± 4.08	21.84 ± 4.24	22.09 ± 4.47	0.569
Left atrial diameter, Mean ± SD	42.86 ± 7.08	41.76 ± 5.99	43.42 ± 7.89	43.77 ± 6.52	0.043
Left ventricular end-systolic diameter (mm), Mean ± SD	28.56 ± 4.28	28.33 ± 3.57	28.65 ± 4.65	28.85 ± 4.66	0.652
Left ventricular end-diastolic diameter (mm), Mean ± SD	43.94 ± 5.35	43.58 ± 4.53	44.08 ± 5.89	44.38 ± 5.36	0.511
Mitral inflow E wave to A wave ratio, M (Q_1_, Q_3_)	0.80 (0.67, 1.38)	0.75 (0.67, 1.25)	0.82 (0.67, 1.40)	0.88 (0.69, 1.50)	0.161
Mitral E wave to tissue Doppler e ratio, M (Q_1_, Q_3_)	0.17 (0.13, 0.23)	0.18 (0.14, 0.23)	0.16 (0.12, 0.23)	0.16 (0.12, 0.20)	0.029
Left ventricular ejection fraction (%), Mean ± SD	64.30 ± 5.83	64.43 ± 5.04	64.33 ± 6.17	63.89 ± 6.51	0.817
Left ventricular apical aneurysm, *n* (%)					0.151
No	429 (98.17)	157 (98.13)	210 (99.06)	62 (95.38)	
Yes	8 (1.83)	3 (1.88)	2 (0.94)	3 (4.62)	
Treatment strategy, *n* (%)					0.104
No treatment	370 (84.67)	126 (78.75)	185 (87.26)	59 (90.77)	
Intervention only	25 (5.72)	12 (7.50)	10 (4.72)	3 (4.62)	
Surgery only	42 (9.61)	22 (13.75)	17 (8.02)	3 (4.62)	
Implantable cardioverter-defibrillator, *n* (%)					0.783
No	423 (96.80)	156 (97.50)	204 (96.23)	63 (96.92)	
Yes	14 (3.20)	4 (2.50)	8 (3.77)	2 (3.08)	
Beta-blockers, *n* (%)					0.944
No	63 (14.42)	22 (13.75)	31 (14.62)	10 (15.38)	
Yes	374 (85.58)	138 (86.25)	181 (85.38)	55 (84.62)	
Calcium channel blockers, *n* (%)					0.714
No	222 (50.80)	82 (51.25)	110 (51.89)	30 (46.15)	
Yes	215 (49.20)	78 (48.75)	102 (48.11)	35 (53.85)	
ACEI/ARB, *n* (%)					0.697
No	272 (62.24)	103 (64.38)	131 (61.79)	38 (58.46)	
Yes	165 (37.76)	57 (35.63)	81 (38.21)	27 (41.54)	
Diuretics, *n* (%)					0.908
No	295 (67.51)	106 (66.25)	145 (68.40)	44 (67.69)	
Yes	142 (32.49)	54 (33.75)	67 (31.60)	21 (32.31)	
Antiarrhythmic drugs, *n* (%)					0.497
No	384 (87.87)	144 (90.00)	185 (87.26)	55 (84.62)	
Yes	53 (12.13)	16 (10.00)	27 (12.74)	10 (15.38)	
Mineralocorticoid receptor antagonists, *n* (%)					0.961
No	281 (64.30)	104 (65.00)	136 (64.15)	41 (63.08)	
Yes	156 (35.70)	56 (35.00)	76 (35.85)	24 (36.92)	
Antiplatelet therapy, *n* (%)					0.898
No	209 (47.83)	78 (48.75)	99 (46.70)	32 (49.23)	
Yes	228 (52.17)	82 (51.25)	113 (53.30)	33 (50.77)	
Anticoagulant therapy, *n* (%)					0.930
No	320 (73.23)	116 (72.50)	157 (74.06)	47 (72.31)	
Yes	117 (26.77)	44 (27.50)	55 (25.94)	18 (27.69)	
Statins, *n* (%)					0.629
No	145 (33.18)	57 (35.63)	69 (32.55)	19 (29.23)	
Yes	292 (66.82)	103 (64.38)	143 (67.45)	46 (70.77)	
SCD					0.515
No	436 (99.77)	159 (99.38)	212 (100.00)	65 (100.00)	
Yes	1 (0.23)	1 (0.63)	0 (0.00)	0 (0.00)	
Hospitalization for heart failure					0.009
No	286 (65.45)	118 (73.75)	133 (62.74)	35 (53.85)	
Yes	151 (34.55)	42 (26.25)	79 (37.26)	30 (46.15)	
New-onset stroke					0.062
No	430 (98.40)	160 (100.00)	207 (97.64)	63 (96.92)	
Yes	7 (1.60)	0 (0.00)	5 (2.36)	2 (3.08)	
Sustained ventricular tachycardia or ventricular fibrillation					0.644
No	435 (99.54)	160 (100.00)	210 (99.06)	65 (100.00)	
Yes	2 (0.46)	0 (0.00)	2 (0.94)	0 (0.00)	
All-cause mortality					0.741
No	424 (97.03)	156 (97.50)	204 (96.23)	64 (98.46)	
Yes	13 (2.97)	4 (2.50)	8 (3.77)	1 (1.54)	
MACE, *n* (%)					0.011
No	274 (62.70)	114 (71.25)	126 (59.43)	34 (52.31)	
Yes	163 (37.30)	46 (28.75)	86 (40.57)	31 (47.69)	
Follow-up time (days), M (Q_1_, Q_3_)	741.00 (359.00, 1,039.00)	780.50 (400.00, 1,021.00)	729.00 (339.00, 1,075.00)	672.00 (345.00, 1,039.00)	0.546

HCM, hypertrophic cardiomyopathy; BMI, body mass index; SCD, sudden cardiac death; NYHA, New York Heart Association grade; WBC, white blood cell; ALT, alanine aminotransferase; AST, aspartate aminotransferase; PCOP, plasma colloid osmotic pressure; eGFR, estimate glomerular filtration rate; HDL-C, high-density lipoprotein cholesterol; LDL-C, low-density lipoprotein cholesterol; NT-proBNP, N-terminal pro-B-type natriuretic peptide; INR, international normalized ratio; ACEI/ARB, angiotensin-converting enzyme inhibitors/angiotensin II receptor blockers; MACE, major adverse cardiovascular events.

### Association between GGT levels and MACE risk among patients with HCM

Table 2 shows the relationship between GGT levels and the risk of MACE. Patients with GGT levels ≥57 U/L [HR = 1.68 (95%CI: 1.06–2.65)] had a higher risk of MACE than those with GGT levels <20 U/L, but no significant association was observed in those with GGT levels of 20–57 U/L (*P* = 0.116). Following adjustment for confounders (model 2), both the GGT ≥57 U/L group [HR = 2.29 (95%CI: 1.30–4.03)] and the GGT 20–57 U/L group [HR = 1.54 (95%CI: 1.06–2.24)] were associated with an increased risk of MACE relative to the reference group (GGT <20 U/L). After adjusting for all clinically significant variables (model 3), both the GGT ≥57 U/L group [HR = 2.29 (95%CI: 1.30–4.03)] and the GGT 20–57 U/L group [HR = 1.72 (95%CI: 1.13–2.62)] were still associated with a higher risk of MACE. Since the majority of MACE events consisted of hospitalizations for heart failure, the association between GGT levels and the risk of hospitalization for heart failure was assessed. GGT levels ≥57 U/L [HR = 2.56 (95%CI: 1.43–4.58)] were linked to a higher risk of hospitalization for heart failure, whereas the association between GGT levels of 20–57 U/L and the risk of hospitalization for heart failure was marginally significant [HR = 1.54 (95%CI: 0.99–2.39), *P* = 0.054] (Supplementary Table S3).

**Table 2:** The relationship between GGT levels and the risk of MACE.

Variables	Model 1		Model 2		Model 3	
	HR (95%CI)	*P*	HR (95%CI)	*P*	HR (95%CI)	*P*
Gamma-Glutamyl Transferase (U/L)						
<20	Ref		Ref		Ref	
20–57	1.34 (0.93–1.92)	0.116	1.54 (1.06–2.24)	0.024	1.72 (1.13–2.62)	0.011
≥57	1.68 (1.06–2.65)	0.026	2.08 (1.29–3.35)	0.002	2.29 (1.30–4.03)	0.004

GGT, gamma-glutamyl transferase; MACE, major adverse cardiovascular events; HR, hazard ratio; CI, confidence interval; Ref, reference;.

Model 1 is a univariate Cox regression model;.

Model 2 is a multivariate Cox regression model that adjusted for age, history of cardiac surgery, alkaline phosphatase, eGFR, non-sustained ventricular tachycardia, and implantable cardioverter-defibrillator;.

Model 3 is a multivariate Cox regression model that adjusted for age, history of cardiac surgery, alkaline phosphatase, eGFR, non-sustained ventricular tachycardia, implantable cardioverter-defibrillator, HCM subtype, left ventricular ejection fraction, NT-proBNP, maximum left ventricular wall thickness, left atrial diameter, family history of SCD, non-sustained ventricular tachycardia, unexplained syncope within the past 6 months, history of HCM-related surgery (all clinically significant variables).

**Figure 2:**
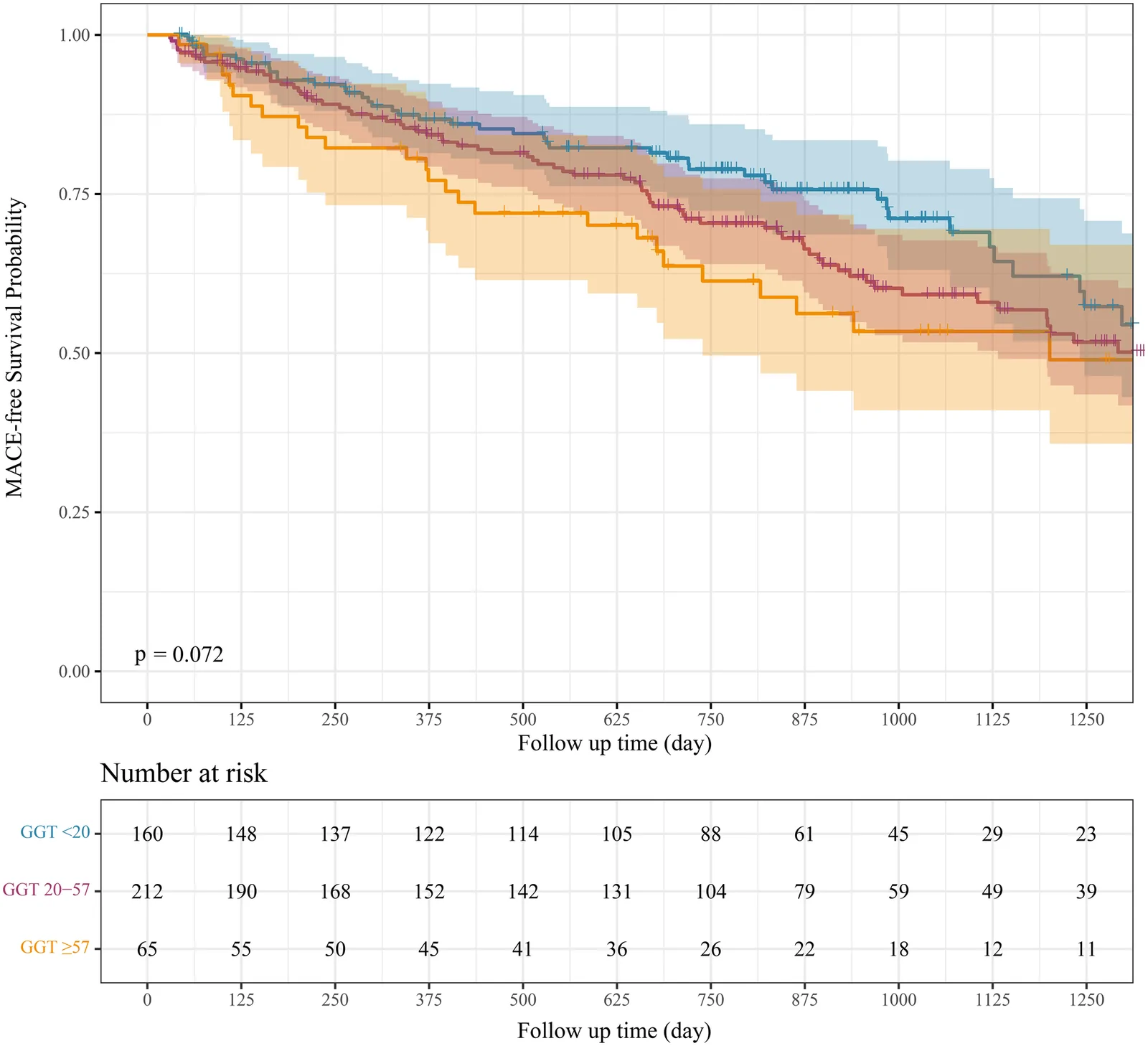
The Kaplan–Meier curve of the probability of MACE-free survival among patients in different GGT level groups. GGT, gamma-glutamyl transferase; MACE, major adverse cardiovascular events.

The Kaplan–Meier curve indicated that the probability of MACE-free survival among patients in different GGT level groups gradually decreases as the follow-up period extends (Figure 2). Overall, there is a trend indicating that the higher the GGT level, the lower the probability of MACE-free survival.

The associations between GGT levels and MACE risk in different subgroups are listed in Table 3. The findings of HCM subtype-based analysis demonstrated that GGT levels of 20–57 U/L [HR = 2.25 (95%CI: 1.15–4.37)] and ≥57 U/L [HR = 3.66 (95%CI: 1.44–9.28)] were both associated with an increased risk of MACE in those with non-obstructive HCM, whereas no significant association was detected in those with resting obstructive HCM (20–57 U/L: *P* = 0.138; ≥57 U/L: *P* = 0.187). However, due to the small sample size, it was not possible to draw valid findings regarding the association between GGT levels and the risk of MACE in those with latent obstructive HCM. NYHA grade-based analysis revealed that GGT levels of 20–57 U/L [HR = 1.78 (95%CI: 1.07–2.97)] and ≥57 U/L [HR = 2.97 (95%CI: 1.51–5.83)] were both linked to an elevated risk of MACE in patients with NYHA grade I/II, while no significant relationship was found in patients with NYHA grade III/IV (20–57 U/L: *P* = 0.319; ≥57 U/L: *P* = 0.131).

**Table 3:** Subgroup analyses of the association between GGT levels and MACE risk.

Subgroups	Variables	Model 1		Model 2		Model	
		HR (95%CI)	*P*	HR (95%CI)	*P*	HR (95%CI)	*P*
HCM subtype	Non-obstructive (*n* = 207)						
	GGT (U/L)						
	<20	Ref		Ref		Ref	
	20–57	1.51 (0.89–2.56)	0.131	1.58 (0.91–2.74)	0.103	2.25 (1.15–4.37)	0.017
	≥57	2.07 (1.10–3.88)	0.024	2.37 (1.24–4.54)	0.009	3.66 (1.44–9.28)	0.006
	Resting obstructive (*n* = 194)						
	GGT (U/L)						
	<20	Ref		Ref		Ref	
	20–57	1.23 (0.72–2.10)	0.452	1.55 (0.87–2.76)	0.134	1.64 (0.85–3.16)	0.138
	≥57	1.82 (0.88–3.73)	0.105	2.31 (1.08–4.90)	0.030	1.94 (0.73–5.19)	0.187
	Latent obstructive (*n* = 36)						
	GGT (U/L)						
	<20	Ref		Ref		Ref	
	20–57	0.93 (0.22–3.90)	0.918	0.69 (0.14–3.35)	0.646	-	-
	≥57	0.29 (0.03–3.06)	0.305	0.14 (0.01–2.37)	0.174	-	-
NYHA grade	Grade I/II (*n* = 340)						
	GGT (U/L)						
	<20	Ref		Ref		Ref	
	20–57	1.17 (0.78–1.76)	0.436	1.33 (0.87–2.04)	0.186	1.78 (1.07–2.97)	0.027
	≥57	1.41 (0.84–2.35)	0.190	1.76 (1.03–3.00)	0.038	2.97 (1.51–5.83)	0.002
	Grade III/IV (*n* = 97)						
	GGT (U/L)						
	<20	Ref		Ref		Ref	
	20–57	2.13 (0.91–4.98)	0.080	2.74 (1.13–6.62)	0.025	2.40 (0.43–13.44)	0.319
	≥57	4.02 (1.39–11.65)	0.010	5.01 (1.58–15.93)	0.006	4.89 (0.62–38.49)	0.131

GGT, gamma-glutamyl transferase; HCM, hypertrophic cardiomyopathy; MACE, major adverse cardiovascular events; NYHA, New York Heart Association grade; HR, hazard ratio; CI, confidence interval; Ref, reference;.

Model 1 is a univariate Cox regression model;.

Model 2 is a multivariate Cox regression model that adjusted for age, history of cardiac surgery, alkaline phosphatase, eGFR, non-sustained ventricular tachycardia, and implantable cardioverter-defibrillator;.

Model 3 is a multivariate Cox regression model that adjusted for age, history of cardiac surgery, alkaline phosphatase, eGFR, non-sustained ventricular tachycardia, implantable cardioverter-defibrillator, HCM subtype, left ventricular ejection fraction, NT-proBNP, maximum left ventricular wall thickness, left atrial diameter, family history of SCD, non-sustained ventricular tachycardia, unexplained syncope within the past 6 months, history of HCM-related surgery (all clinically significant variables).

**Figure 3:**
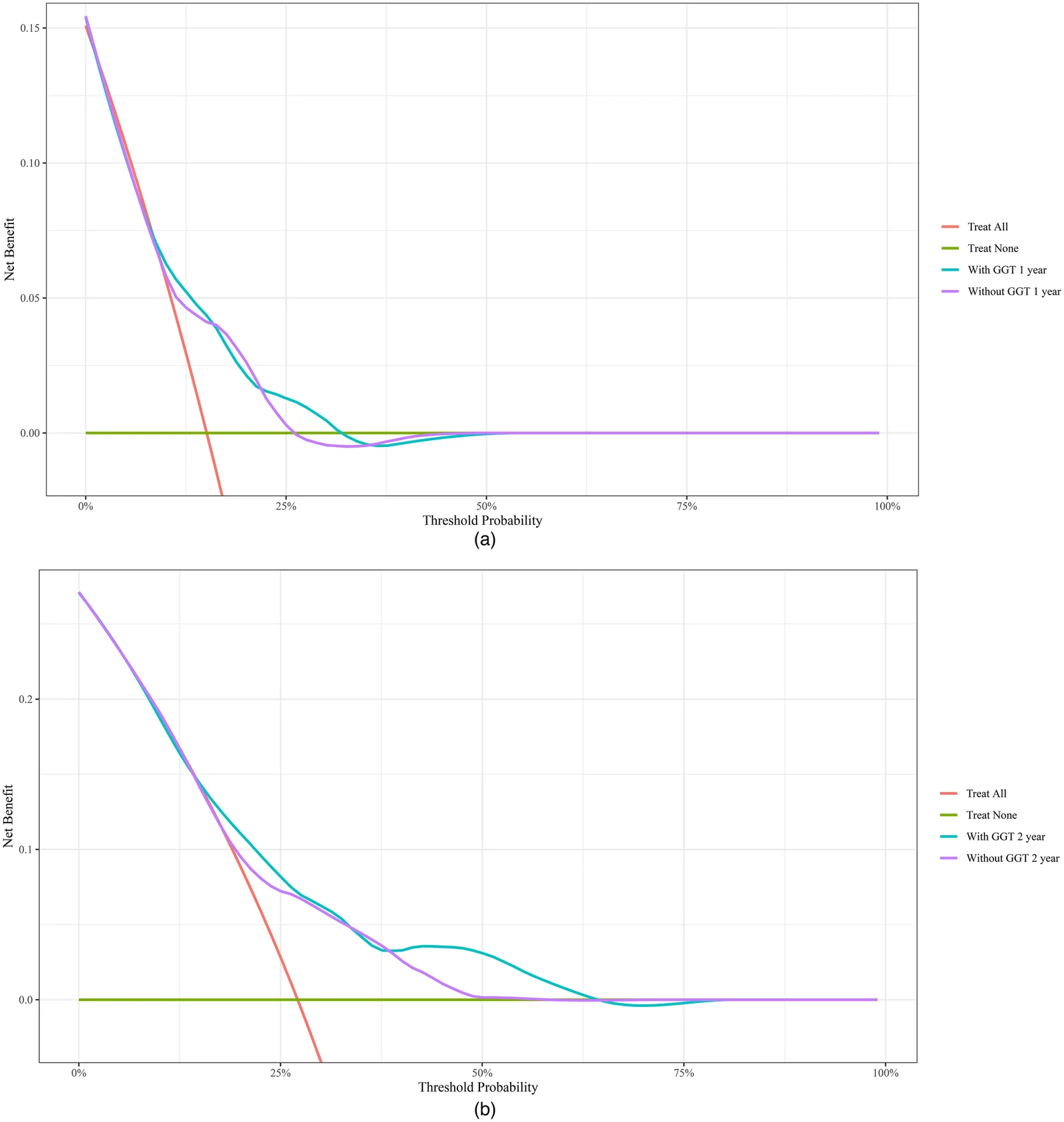
The decision curve analysis (DCA) of the predictive value of GGT levels for MACE risk. **(a)** DCA for 1-year MACE risk; **(b)** DCA for 2-year MACE risk. GGT, gamma-glutamyl transferase; MACE, major adverse cardiovascular events.

Table 4 presents the predictive performance of the Cox model for MACE risk. The model without GGT inclusion had an AUC of 0.656 (95%CI: 0.576–0.736) for 1-year MACE risk prediction and 0.653 (95%CI: 0.587–0.719) for 2-year MACE risk prediction. When GGT was incorporated into the model, the AUC for predicting 1-year MACE risk was 0.671 (95%CI: 0.594–0.748), and the AUC for 2-year MACE risk prediction was 0.683 (95%CI: 0.620–0.746). The model with GGT incorporated had an NRI of 0.330 (95%CI: 0.106–0.618) and an IDI of 0.0256 (95%CI: 0.0057–0.0649) for 2-year MACE risk prediction. These findings suggested that incorporating the GGT indicator into the established Cox model can slightly enhance the model's risk stratification capacity and overall discriminatory performance. The decision curve analysis indicated that after the addition of GGT, the model exhibited a slight improvement in net benefits across different risk thresholds (Figure 3). In addition, a comparison of the predictive performance of GGT, HCM risk-SCD, and NT-proBNP for MACE risk was analyzed. The AUC values for 2-year MACE risk prediction were 0.574 (95%CI: 0.514–0.635) for GGT, 0.530 (95%CI: 0.463–0.598) for HCM risk-SCD, and 0.533 (95%CI: 0.498–0.568) for NT-proBNP (Supplementary Table S4). The Delong test indicated that no statistically significant differences were found among these AUCs (*P* > 0.05).

**Table 4:** The predictive performance of the Cox model for MACE risk.

Model	1-year AUC (95%CI)	2-year AUC (95%CI)	1-year NRI (95%CI)	2-year NRI (95%CI)	1-year IDI (95%CI)	2-year IDI (95%CI)
Without GGT	0.656 (0.576–0.736)	0.653 (0.587–0.719)	-	-	-	-
With GGT	0.671 (0.594–0.748)	0.683 (0.620–0.746)	0.187 (−0.034–0.538)	0.330 (0.106–0.618)	0.0,118 (0.0002–0.0410)	0.0256 (0.0057–0.0649)

GGT, gamma-glutamyl transferase; MACE, major adverse cardiovascular events; CI, confidence interval; AUC, the area under the curve; NRI, net reclassification index; IDI, integrated discrimination improvement.

The Cox regression model included age, history of cardiac surgery, alkaline phosphatase, eGFR, non-sustained ventricular tachycardia, and implantable cardioverter-defibrillator.

Sensitivity analysis that excluded patients with an implantable cardioverter-defibrillator demonstrated that the GGT levels of 20–57 U/L [HR = 1.82 (95%CI: 1.18–2.79)] and ≥57 U/L [HR = 2.69 (95%CI: 1.52–4.76)] remained associated with a higher risk of MACE (Supplementary Table S5).

## Discussion

This research explored the association between GGT levels and MACE risk among patients with HCM. Patients with GGT ≥57 U/L and GGT 20–57 U/L were associated with a higher risk of MACE than those with GGT <20 U/L. Moreover, GGT levels of 20–57 U/L and ≥57 U/L were related to an increased risk of MACE in patients with non-obstructive HCM, whereas the association was non-significant for resting obstructive HCM and latent obstructive HCM. In terms of the predictive value of GGT, the inclusion of GGT resulted in only a slight improvement in the Cox regression model's ability to predict MACE risk.

HCM is a sarcomeric disorder in which genetic defects in sarcomere proteins lead to left ventricular hypertrophy, excessive contraction, and diastolic dysfunction (2). The cardiac cycle (systole-diastole) involves the binding of actin and myosin in cardiomyocytes (27). This binding subsequently causes a conformational change in myosin, thereby triggering the power stroke (systole). Genetic defects in sarcomere proteins ultimately affect biomechanical stress sensing, bioenergetics, and calcium (Ca²⁺) cycling/sensitivity, promoting cardiac muscle cell reorganization and disorganization, leading to fibrosis and asymmetric septal hypertrophy (28). MACE exhibits a high prevalence among individuals with HCM. Among those diagnosed with HCM before 40 years of age, 90% will experience MACE during their lifetime, 70% of whom will develop atrial fibrillation and 65% will exhibit signs of heart failure (6). Our findings demonstrated that the incidence of MACE among current HCM patients was 37.30%. Moreover, we explored the association between GGT levels and MACE risk among patients with HCM. GGT is a widely distributed enzyme involved in the metabolism of glutathione, which is the primary physiological antioxidant in humans. Elevated serum GGT levels are frequently regarded as an indirect indicator of enhanced oxidative stress (29). Our findings revealed that high GGT levels were associated with a higher risk of MACE. Previous studies have demonstrated that elevated GGT levels were linked to an increased risk of heart failure, SCD, cardiovascular events, atherosclerosis, and mortality (17, 19, 30, 31). In addition, our findings found that high GGT levels were linked to a higher risk of MACE in those with non-obstructive HCM. For resting obstructive HCM, an association between GGT levels and the risk of MACE was observed after adjusting for some covariates (model 2). However, this association was no longer significant after further adjustment for all clinically relevant variables (model 3). The loss of statistical significance may be attributable to adequate adjustment for confounding factors or insufficient statistical power. Given the small sample size and low number of events, adjusting for a large number of clinically relevant variables inevitably increased the complexity of the model and reduced statistical power, leading to larger standard errors and wider confidence intervals. Although statistical significance was lost, the direction and magnitude of the effect estimates remained largely consistent, suggesting that this result may be due to insufficient precision in the estimates rather than a complete lack of association. These results should be interpreted with caution and require validation in larger cohorts. Due to the small sample size, it was not possible to draw valid conclusions in patients with latent obstructive HCM. Some studies have shown that in patients with symptomatic latent obstructive HCM, the degree of septal hypertrophy is less severe than that in patients with resting obstructive HCM (32, 33). Septal hypertrophy may not be the sole cause of LVOT obstruction among patients with latent obstructive HCM. The varying associations between GGT levels and MACE risk across different HCM subtypes deserve further investigation.

The link between elevated GGT levels and an increased MACE risk in patients with HCM may be attributed to the effect of GGT on specific subtypes of MACE. The mechanisms linking elevated GGT levels to an increased risk of heart failure and arrhythmias include pro-oxidant and pro-inflammatory activities, as well as the direct role of GGT in atherosclerotic plaque formation (11, 34). GGT serves as a marker of increased oxidative stress, which contributes to ventricular remodeling and endothelial dysfunction-key factors in the pathogenesis of heart failure (35). Active GGT influences plaque formation and its progression to vulnerable plaques (34, 36). By participating in atherosclerotic plaque formation, GGT may also trigger acute myocardial ischemia, the most common trigger for fatal arrhythmias (30, 34). Elevated GGT levels may also reflect the degree of inflammation, which is a critical pathophysiological mechanism in atherosclerosis (37). Systemic inflammation induces increased endothelial expression of intercellular adhesion molecules. These molecules lead to oxidative stress, impair myocardial microcirculation, and induce myocardial cell stiffness, hypertrophy, apoptosis, or necrosis, ultimately resulting in left ventricular remodeling (19, 38). The impact of inflammation on SCD is also likely mediated through coronary atherosclerotic lesions and vulnerable plaques (11, 39). Elevated GGT levels are linked to metabolic syndrome, obesity, and insulin resistance (20, 21). GGT contributes to the impairment of the antioxidant defense system and promotes oxidative stress, as well as the potential development of fatty liver disease, which is also related to insulin resistance and oxidative stress (11). Insulin resistance is considered a potential mechanism linking GGT to metabolic syndrome (37). Systemic inflammation, endothelial dysfunction, insulin resistance, oxidative stress, and lipid metabolism abnormalities are all mechanisms underlying cardiovascular disease and increased mortality (40–42).

This study evaluated the association between GGT levels and MACE risk in a well-defined HCM cohort, rather than in the general population or in patients with heart failure or arrhythmias of various etiologies. We not only assessed the independent association between GGT and MACE risk but also used time-dependent AUC, NRI, IDI, and decision curve analysis to evaluate its incremental predictive value beyond traditional clinical variables. Although the predictive performance of the model improved after incorporating GGT, the increase in AUC was small, particularly for predicting 1-year outcomes. Accordingly, the results of this study do not yet demonstrate a clear clinical benefit of GGT, nor are they sufficient to support its use as an independent biomarker in clinical practice. The clinical utility of GGT still requires further evaluation through external validation and clinical utility analysis. Nevertheless, several limitations should be noted. First, this study was designed as a single-center retrospective cohort study, which may be subject to selection bias and information bias, potentially compromising the accuracy of the study findings. Second, due to the lack of relevant inspections and records, some potential confounding factors, including genetic information and dietary information, cannot be taken into account and controlled. Third, this study only evaluated the association between a single measurement of GGT levels and MACE risk. The correlation between long-term GGT trajectories, GGT variability, and MACE risk warrants further analysis. Fourth, the GGT cutoff values were derived using an outcome-driven optimization method (X-title) applied to the cohort in this study and have not yet been validated externally. These thresholds may be influenced by cohort-specific characteristics and should not be regarded as established clinical cutoff values. Their reproducibility needs to be validated in an independent population with HCM. Fifth, part of the follow-up information in this study was collected via telephone interviews, and not all reported events could be fully verified through complete inpatient medical records or dynamic electrocardiographic monitoring. This may introduce recall bias and lead to the underestimation of outcome events. Specifically, persistent ventricular tachycardia that does not result in hospitalization, presents with atypical symptoms, or lacks objective rhythm recordings may be unrecognized and missed during follow-up.

## Conclusions

GGT levels of 20–57 U/L and ≥57 U/L were linked to an increased risk of MACE than those with GGT <20 U/L among patients with HCM. For the subtypes of HCM, the association between GGT levels and MACE risk was significant in patients with non-obstructive HCM. Future research may need to further explore the prognostic value of GGT levels in patients with HCM.

## Data Availability

The original contributions presented in the study are included in the article/Supplementary Material, further inquiries can be directed to the corresponding author.
