# Supplementary material for: Association between gamma-glutamyl transferase and risk of major adverse cardiovascular events in patients with hypertrophic cardiomyopathy: a retrospective cohort study
Source: Front Cardiovasc Med. 2026 Aug 28;13:1900684. doi: 10.3389/fcvm.2026.1900684 (PMC13562296; doi:10.3389/fcvm.2026.1900684)
Supplement: Supplementary file 1 [file Datasheet1.docx]

**Supplementary Table 1**. Difference comparison between pre-imputation and post-imputation datasets.

| Variables | Proportion of missing, n (%) | Post-imputation | Pre-imputation | Statistics | *P* |
| --- | --- | --- | --- | --- | --- |
| WBC count (10^9^/L), Mean ± SD | 2 (0.46%) | 6.21 ± 1.77 | 6.21 ± 1.77 | t=-0.010 | 0.992 |
| Lymphocyte count (10^9^/L), M (Q_1_, Q_3_) | 2 (0.46%) | 1.70 (1.30, 2.20) | 1.70 (1.30, 2.20) | Z=0.004 | 0.997 |
| Hemoglobin (g/L), M (Q_1_, Q_3_) | 2 (0.46%) | 141.00 (127.00, 152.00) | 141.00 (127.00, 152.00) | Z=-0.054 | 0.957 |
| Platelet count (10^9^/L), Mean ± SD | 2 (0.46%) | 201.24 ± 59.04 | 201.17 ± 59.16 | t=0.018 | 0.986 |
| PCOP (mOsm/L), Mean ± SD | 4 (0.92%) | 13.77 ± 2.95 | 13.76 ± 2.95 | t=0.057 | 0.955 |
| Anion gap (mmol/L), Mean ± SD | 4 (0.92%) | 300.72 ± 12.17 | 300.69 ± 12.21 | t=0.037 | 0.971 |
| Glucose (mmol/L), M (Q_1_, Q_3_) | 3 (0.69%) | 4.80 (4.30, 5.50) | 4.80 (4.30, 5.50) | Z=-0.040 | 0.968 |
| eGFR (mL/min), M (Q_1_, Q_3_) | 49 (11.21%) | 83.00 (71.00, 95.40) | 83.00 (71.00, 96.00) | Z=0.077 | 0.939 |
| Triglyceride (mmol/L), M (Q_1_, Q_3_) | 18 (4.12%) | 1.30 (0.95, 1.90) | 1.30 (0.90, 1.90) | Z=-0.080 | 0.936 |
| Total Cholesterol (mmol/L), Mean ± SD | 18 (4.12%) | 3.93 ± 0.97 | 3.91 ± 0.96 | t=0.269 | 0.788 |
| HDL-C (mmol/L), M (Q_1_, Q_3_) | 19 (4.35%) | 1.00 (0.80, 1.20) | 1.00 (0.80, 1.20) | Z=-0.256 | 0.798 |
| LDL-C (mmol/L), M (Q_1_, Q_3_) | 20 (4.58%) | 2.30 (1.70, 2.90) | 2.30 (1.70, 2.90) | Z=-0.285 | 0.776 |
| NT-proBNP (pg/mL), M (Q_1_, Q_3_) | 56 (12.81%) | 795.00 (299.00, 1823.00) | 795.00 (300.00, 1783.00) | Z=0.069 | 0.945 |
| INR, Mean ± SD | 7 (1.60%) | 0.99 ± 0.29 | 0.99 ± 0.30 | t=-0.014 | 0.989 |
| Fibrinogen (g/L), M (Q_1_, Q_3_) | 8 (1.83%) | 2.60 (2.20, 3.10) | 2.60 (2.20, 3.10) | Z=-0.018 | 0.986 |
| BMI (kg/m^2^), Mean ± SD | 108 (24.71%) | 26.72 ± 3.79 | 26.69 ± 3.83 | t=0.102 | 0.918 |
| Mitral inflow E wave to A wave ratio, M (Q_1_, Q_3_) | 52 (11.90%) | 0.80 (0.67, 1.38) | 0.78 (0.67, 1.33) | Z=-0.467 | 0.641 |
| Mitral E wave to tissue Doppler e ratio, M (Q_1_, Q_3_) | 132 (30.21%) | 0.17 (0.13, 0.23) | 0.17 (0.13, 0.23) | Z=-0.191 | 0.848 |
| Non-sustained ventricular tachycardia, n (%) | 2 (0.46%) |  |  | χ^2^=0.000 | 0.991 |
| No |  | 424 (97.03) | 422 (97.01) |  |  |
| Yes |  | 13 (2.97) | 13 (2.99) |  |  |

Note: WBC, white blood cell; PCOP, plasma colloid osmotic pressure; eGFR, estimate glomerular filtration rate; HDL-C, high-density lipoprotein cholesterol; LDL-C, low-density lipoprotein cholesterol; NT-proBNP, N-terminal pro-B-type natriuretic peptide; INR, international normalized ratio; BMI, body mass index.

**Supplementary Table 2**. Screening of confounding factors related to the risk of MACE.

| Variables | Univariate Cox regression model | |
| --- | --- | --- |
|  | HR (95%CI) | P |
| Age | 1.02 (1.01-1.03) | 0.013 |
| Gender |  |  |
| Male | Ref |  |
| Female | 1.20 (0.87-1.64) | 0.265 |
| BMI (kg/m^2^) |  |  |
| <24 | Ref |  |
| ≥24 | 1.01 (0.69-1.47) | 0.959 |
| Smoking status |  |  |
| No | Ref |  |
| Quit smoking | 0.99 (0.67-1.46) | 0.967 |
| Smoking now | 1.07 (0.74-1.54) | 0.733 |
| Drinking status |  |  |
| No | Ref |  |
| Quit drinking | 1.13 (0.79-1.63) | 0.495 |
| Drinking now | 0.92 (0.62-1.38) | 0.691 |
| Time from current hospital visit to diagnosis | 1.00 (1.00-1.00) | 0.303 |
| Family history of hypertrophic cardiomyopathy |  |  |
| No | Ref |  |
| Yes | 1.47 (0.75-2.89) | 0.261 |
| Family history of sudden cardiac death |  |  |
| No | Ref |  |
| Yes | 1.64 (0.52-5.16) | 0.394 |
| Unexplained syncope within the past 6 months |  |  |
| No | Ref |  |
| Yes | 1.47 (0.90-2.40) | 0.126 |
| History of cardiac surgery |  |  |
| No | Ref |  |
| Yes | 1.80 (1.32-2.47) | <.001 |
| History of HCM-related surgery |  |  |
| No | Ref |  |
| Yes | 1.42 (0.95-2.14) | 0.090 |
| Chest tightness |  |  |
| No | Ref |  |
| Yes | 1.07 (0.78-1.46) | 0.679 |
| Palpitations |  |  |
| No | Ref |  |
| Yes | 1.22 (0.86-1.74) | 0.260 |
| Chest pain |  |  |
| No | Ref |  |
| Yes | 1.02 (0.64-1.63) | 0.942 |
| Hypertension |  |  |
| No | Ref |  |
| Yes | 1.11 (0.81-1.51) | 0.513 |
| Diabetes |  |  |
| No | Ref |  |
| Yes | 1.28 (0.75-2.18) | 0.361 |
| Dyslipidemia |  |  |
| No | Ref |  |
| Yes | 1.37 (0.70-2.69) | 0.365 |
| Coronary heart disease |  |  |
| No | Ref |  |
| Yes | 1.23 (0.90-1.68) | 0.201 |
| Atrial fibrillation |  |  |
| No | Ref |  |
| Yes | 1.35 (0.95-1.91) | 0.092 |
| NYHA grade |  |  |
| Grade I/II | Ref |  |
| Grade III/IV | 1.17 (0.81-1.69) | 0.411 |
| WBC count | 1.02 (0.93-1.11) | 0.706 |
| Lymphocyte count | 0.79 (0.61-1.02) | 0.071 |
| Hemoglobin | 1.00 (1.00-1.00) | 0.421 |
| Platelet count | 1.00 (1.00-1.00) | 0.473 |
| ALT | 1.00 (1.00-1.01) | 0.363 |
| AST | 1.01 (1.00-1.02) | 0.051 |
| Albumin | 0.99 (0.96-1.02) | 0.553 |
| Total Bilirubin | 1.01 (0.99-1.03) | 0.337 |
| Alkaline Phosphatase | 1.01 (1.01-1.02) | 0.021 |
| Anion gap | 1.00 (0.99-1.02) | 0.753 |
| PCOP | 0.99 (0.94-1.05) | 0.811 |
| Glucose | 0.98 (0.93-1.04) | 0.483 |
| eGFR | 0.99 (0.99-0.99) | 0.030 |
| Triglyceride | 0.99 (0.87-1.13) | 0.891 |
| Total Cholesterol | 1.02 (0.87-1.19) | 0.786 |
| HDL-C | 0.90 (0.66-1.23) | 0.516 |
| LDL-C | 0.96 (0.80-1.15) | 0.660 |
| NT-proBNP | 1.00 (1.00-1.00) | 0.096 |
| INR | 0.80 (0.43-1.49) | 0.483 |
| Fibrinogen | 1.03 (0.95-1.11) | 0.437 |
| Non-sustained ventricular tachycardia |  |  |
| No | Ref |  |
| Yes | 2.52 (1.24-5.15) | 0.011 |
| Hypertrophic cardiomyopathy subtype |  |  |
| Non-obstructive | Ref |  |
| Obstructive | 1.21 (0.87-1.67) | 0.253 |
| Latent obstructive | 0.82 (0.42-1.58) | 0.545 |
| Maximum left ventricular wall thickness | 0.98 (0.94-1.02) | 0.241 |
| Left atrial diameter | 1.00 (0.98-1.02) | 0.836 |
| Left ventricular end-systolic diameter | 1.00 (0.96-1.03) | 0.809 |
| Left ventricular end-diastolic diameter | 1.00 (0.97-1.03) | 0.950 |
| Mitral inflow E wave to A wave ratio | 0.97 (0.71-1.32) | 0.843 |
| Mitral E wave to tissue Doppler e ratio | 2.05 (0.75-5.60) | 0.160 |
| Left ventricular ejection fraction | 0.99 (0.96-1.02) | 0.431 |
| Left ventricular apical aneurysm |  |  |
| No | Ref |  |
| Yes | 1.75 (0.72-4.27) | 0.221 |
| Treatment strategy |  |  |
| No treatment | 0.65 (0.35-1.20) | 0.164 |
| Intervention only | Ref |  |
| Surgery only | 0.38 (0.13-1.10) | 0.073 |
| Implantable cardioverter-defibrillator |  |  |
| No | Ref |  |
| Yes | 2.23 (1.09-4.55) | 0.027 |
| Beta-blockers |  |  |
| No | Ref |  |
| Yes | 1.40 (0.85-2.31) | 0.191 |
| Calcium channel blockers |  |  |
| No | Ref |  |
| Yes | 0.91 (0.66-1.23) | 0.529 |
| ACEI/ARB |  |  |
| No | Ref |  |
| Yes | 1.12 (0.82-1.53) | 0.475 |
| Diuretics |  |  |
| No | Ref |  |
| Yes | 1.04 (0.75-1.46) | 0.798 |
| Antiarrhythmic drugs |  |  |
| No | Ref |  |
| Yes | 1.43 (0.92-2.21) | 0.108 |
| Mineralocorticoid receptor antagonists |  |  |
| No | Ref |  |
| Yes | 0.95 (0.69-1.32) | 0.764 |
| Antiplatelet therapy |  |  |
| No | Ref |  |
| Yes | 0.83 (0.61-1.13) | 0.231 |
| Anticoagulant therapy |  |  |
| No | Ref |  |
| Yes | 1.22 (0.86-1.73) | 0.269 |
| Statins |  |  |
| No | Ref |  |
| Yes | 1.23 (0.88-1.72) | 0.236 |

Note: MACE, major adverse cardiovascular events; BMI, body mass index; HCM, hypertrophic cardiomyopathy; NYHA, New York Heart Association grade; WBC, white blood cell; ALT, alanine aminotransferase; AST, aspartate aminotransferase; PCOP, plasma colloid osmotic pressure; eGFR, estimate glomerular filtration rate; HDL-C, high-density lipoprotein cholesterol; LDL-C, low-density lipoprotein cholesterol; NT-proBNP, N-terminal pro-B-type natriuretic peptide; INR, international normalized ratio; ACEI/ARB, angiotensin-converting enzyme inhibitors/angiotensin II receptor blockers.

**Supplementary Table 3**. The association between GGT levels and the risk of hospitalization for heart failure.

| Variables | Outcome/Total  (%) | Model 1 | | Model 2 | | Model 3 | |
| --- | --- | --- | --- | --- | --- | --- | --- |
|  |  | HR (95%CI) | P | HR (95%CI) | P | HR (95%CI) | P |
| GGT (U/L) |  |  |  |  |  |  |  |
| <20 | 42/160 (26.25) | Ref |  | Ref |  | Ref |  |
| 20-57 | 79/212 (37.26) | 1.29 (0.88-1.89) | 0.184 | 1.38 (0.93-2.04) | 0.110 | 1.54 (0.99-2.39) | 0.054 |
| ≥57 | 30/65 (46.15) | 1.77 (1.10-2.83) | 0.018 | 1.99 (1.22-3.25) | 0.006 | 2.56 (1.43-4.58) | 0.002 |

Note: GGT, gamma-glutamyl transferase; HR, hazard ratio; CI, confidence interval; Ref, reference;

Model 1 is a univariate Cox regression model;

Model 2 is a multivariate Cox regression model that adjusted for age, history of cardiac surgery, alkaline phosphatase, eGFR, non-sustained ventricular tachycardia, and implantable cardioverter-defibrillator;

Model 3 is a multivariate Cox regression model that adjusted for age, history of cardiac surgery, alkaline phosphatase, eGFR, non-sustained ventricular tachycardia, implantable cardioverter-defibrillator, HCM subtype, left ventricular ejection fraction, NT-proBNP, maximum left ventricular wall thickness, left atrial diameter, family history of SCD, non-sustained ventricular tachycardia, unexplained syncope within the past 6 months, history of HCM-related surgery (all clinically significant variables).

**Supplementary Table 4**. Comparison of predictive performance for MACE risk.

| Model | 1-year AUC (95%CI) | Delong test | 2-year AUC (95%CI) | Delong test |
| --- | --- | --- | --- | --- |
| GGT | 0.538 (0.465−0.610) | Ref | 0.574 (0.514−0.635) | Ref |
| HCM risk-SCD | 0.532 (0.450−0.614) | 0.925 | 0.530 (0.463−0.598) | 0.336 |
| NT-proBNP | 0.548 (0.516−0.581) | 0.804 | 0.533 (0.498−0.568) | 0.263 |

Note: GGT, Gamma-Glutamyl Transferase; MACE, major adverse cardiovascular events; HCM, hypertrophic cardiomyopathy; SCD, sudden cardiac death; NT-proBNP, N-terminal pro-B-type natriuretic peptide; CI, confidence interval; AUC, the area under the curve; Ref, reference;

HCM risk-SCD, a new clinical risk prediction model for	SCD in HCM that has been reported (PMID: 24126876).

**Supplementary Table 5**. Sensitivity analysis of the association between GGT levels and MACE risk (excluded patients with implantable cardioverter-defibrillator).

| Variables | Model 1 | | Model 2 | | Model 3 | |
| --- | --- | --- | --- | --- | --- | --- |
|  | HR (95%CI) | P | HR (95%CI) | P | HR (95%CI) | P |
| Gamma-Glutamyl Transferase (U/L) |  |  |  |  |  |  |
| <20 | Ref |  | Ref |  | Ref |  |
| 20-57 | 1.30 (0.90-1.88) | 0.163 | 1.53 (1.04-2.23) | 0.030 | 1.82 (1.18-2.79) | 0.006 |
| ≥57 | 1.67 (1.05-2.65) | 0.031 | 2.04 (1.26-3.30) | 0.004 | 2.69 (1.52-4.76) | <0.001 |

Note: MACE, major adverse cardiovascular events; HR, hazard ratio; CI, confidence interval; Ref, reference;

Model 1 is a univariate Cox regression model;

Model 2 is a multivariate Cox regression model that adjusted for age, history of cardiac surgery, alkaline phosphatase, eGFR, non-sustained ventricular tachycardia, and implantable cardioverter-defibrillator;

Model 3 is a multivariate Cox regression model that adjusted for age, history of cardiac surgery, alkaline phosphatase, eGFR, non-sustained ventricular tachycardia, implantable cardioverter-defibrillator, HCM subtype, left ventricular ejection fraction, NT-proBNP, maximum left ventricular wall thickness, left atrial diameter, family history of SCD, non-sustained ventricular tachycardia, unexplained syncope within the past 6 months, history of HCM-related surgery (all clinically significant variables).

**Supplementary Figure 1**. The optimal cutoff value for GGT grouping in the X-tile software. GGT, Gamma-Glutamyl Transferase.
